# Away-from-home food during coronavirus pandemic

**DOI:** 10.1017/S1368980020001470

**Published:** 2020-07

**Authors:** Ilana N. Bezerra

**Affiliations:** Ceará State University, Fortaleza, Ceará, Brazil Email: ilana.bezerra@uece.br

Madam,

The pandemic of coronavirus we are facing all around the world is changing our way of life and will undoubtedly bring us many challenges that will suppress socio-economic effects^(1)^. We could number many impacts related to public health nutrition, from hunger, nutritional insecurity to increasing consumption of ultraprocessed foods, development of nutritional disorders and obesity.

However, there is a habit that we cannot overlook in our actual reality: the consumption of away-from-home food (AFHF). The increase in this habit is well recognised in many developed and developing countries. In recent years, new alternatives of access to foods prepared away from home have emerged, such as apps for home-delivery foods. Due to coronavirus outbreak, more people are relying on these food delivery apps and other delivery services^(2)^.

In scientific literature, there is a discussion about the definition of AFHF, whether it should be based on the place where food is prepared or on the place where food is consumed. If we define AFHF based on the place where food is consumed, during the isolation period due to coronavirus pandemic, the majority of people will not eat away from home. However, it is important to consider changes related to the manner food is being ordered during the pandemic. Although we do not have evidence regarding the increase in home food delivery services, many companies are hiring employees to handle food delivery demand^(2–4)^. Therefore, if we define AFHF based on the place food is prepared, we will have a more reliable evaluation of this habit around the world.

The recognition of AFHF as an important contributor to diet and its negative impacts on health calls the attention for a public health issue that nutrition science will experience due to coronavirus pandemic^(5)^. In a public health perspective, we should also consider other issues related to the consumption of food away from home during the pandemic (microbiologic contamination, virus dissemination due to the contact with drivers, traffic accidents, and poor work conditions of cookers and drivers), beyond the nutritional quality of foods produced outside the home. Therefore, the exposure to foods prepared away from home, especially those delivered at home, can bring us many challenges that require intense solution for coping the problems related to AFHF.
